# Differential effects of repeated inspiratory and limb muscle loading on effort perception in patients with obstructive sleep apnea and healthy males

**DOI:** 10.14814/phy2.15732

**Published:** 2023-06-03

**Authors:** Claire Griffith‐Mcgeever, Julian Owen, Christopher Earing, Damian McKeon, Hans‐Peter Kubis

**Affiliations:** ^1^ Cardiorespiratory Department Wrexham Maelor Hospital Wrexham UK; ^2^ School of Human and Behavioural Sciences Bangor University Bangor UK; ^3^ Pulmonary and Sleep Department Ysbyty Gwynedd Hospital Bangor UK

## Abstract

Obstructive sleep apnea (OSA) is characterized by collapse of the upper airways during sleep. The contribution of alterations in effort perception is not understood. This study investigated the response of inspiratory and quadriceps muscles to repetitive loading on effort perception in OSA patients, pre and post continuous positive airway pressure (CPAP) treatment, and in healthy individuals. Twenty‐one OSA patients and 40 healthy participants completed protocols for repetitive inspiratory and leg muscle loading combined with intermittent rating of perceived exertion (RPE 14—*somewhat hard/hard*) to assess effort sensitivity. Electromyography, inspiratory pressure and isometric force were measured. OSA patients reported higher fatiguability of respiratory and leg muscles than controls. OSA patients revealed lower effort sensitivity in the leg muscles compared with controls, while repetitive loading led to a decline in force production. In the respiratory system, OSA patients revealed similar effort sensitivity at baseline compared with controls, but a large reduction in effort sensitivity after loading. Baseline effort sensitivity was correlated with apnea‐hypopnea index (AHI). After CPAP treatment, OSA patients revealed a decreased baseline effort sensitivity with a missing loading response. Effort sensitivity was differentially affected in the respiratory and leg systems with outcomes of CPAP treatment suggesting a full reversibility. Outcomes suggest that reversible adaptive response of effort perception in the respiratory system might contribute to the severity of OSA.

## 1 INTRODUCTION

Obstructive sleep apnea (OSA) is a common breathing disorder characterized by repeated pharyngeal collapse of the upper respiratory airways during sleep resulting in periodic apnea and hypopnea episodes. OSA is linked to many severe health consequences including increased neurocognitive, metabolic, cardiovascular morbidity, and mortality (Jean‐Louis et al., 2008). While distinct physiological and anatomical factors such as skeletal and/or soft tissue abnormalities affecting the mandibular or maxillary size and position, nasal cavities, tonsils and adenoids vary in their contribution to symptomatology (Owens et al., 2015), it has been suggested that ventilatory instability and low arousal threshold in response to obstruction are mainly caused by changes to regulatory respiratory components. In particular, a higher loop gain with altered chemosensitivity, an elevated plant gain, and a reduced dilator muscle recruitment are known to influence OSA severity (Deacon‐Diaz & Malhotra, 2018; Eckert et al., 2013).

Obesity has long been recognized as the major epidemiological risk factor for OSA (Carter & Watenpaugh, 2008; Young et al., 2004). Besides upper airway function which can be compromised by increased deposition of adipose tissue within the pharyngeal walls (Hudgel et al., 1990; Schwartz et al., 2010; Shelton et al., 1993), alterations of mechanical properties of the chest wall and lungs by excessive fat accumulation can cause mass loading of the respiratory muscles (Horner et al., 1989; Koenig & Thach, 1988; Peters & Dixon, 2018). A main feature of OSA is the cyclical pattern of upper airway instability followed by strong inspiratory efforts with arousal, with the possibility of overloading the respiratory muscles (Wilcox et al., 1990). Indeed, Chien et al., (Chien et al., 2010) reported that OSA patients demonstrate reduced inspiratory muscle capacity and increased fatigability in response to loading. Similarly, obese individuals revealed mechanical inefficiency of the respiratory muscles with increased work of breathing and higher fatigability (Cherniack & Guenter, 1961; Lopata & Onal, 1982; Sharp et al., 1986). However, the impact of loading upon the respiratory muscles and its consequences for effort perception is relatively unknown within OSA population.

Effort perception is thought to be directly linked to the size of the motor command generated for a task (Pageaux, 2016). The perception of effort has typically been suggested to be a direct function of collateral discharge of a motor command that is produced for an action and is viewed to have a smaller, supporting influence from afferent feedback via peripheral receptors (Proske & Allen, 2019). Corollary discharge refers to a copy of motor discharge sent to the sensory cortex in order to generate a perceived effort. Centrally mediated sensations are presumed to arise from internal neural correlates (i.e., corollary discharges) of the descending motor command that provide an internal adjustment of the sensory centers and are thought to reflect the magnitude of the voluntary motor command (McCloskey, 1981). Afferent feedback generated within muscle spindles, tendon organs, and pressure‐sensitive skin receptors are also thought to be key to providing and updating the central motor patterns with information regarding the force and effort required to complete a given task (Amann et al., 2010). The complete afferent signal is suggested to have both exafferent and reafferent components (Proske & Allen, 2019). Effort perception is known to be strongly influenced by muscular fatigue and reduced capacity (Jones & Hunter, 1983) and is an important contributing factor to dyspnoea sensation in obstructive conditions (Manning & Mahler, 2001; O'Donnell et al., 2007). While effort is perceived during wakefulness, the underlying mechanisms could also be important for controlling the size of the motor command in response to airway obstructions in OSA which may further influence the arousal from sleep.

A higher negative inspiratory pressure for arousal from sleep was found in OSA patients and changes in the arousal threshold have been associated with OSA severity that is, apnea‐hypopnoea index (AHI), with reversibility after continuous positive airway pressure (CPAP) (Edwards et al., 2014). Moreover, Tun et al., (Tun et al., 2000) revealed that OSA patients have an impaired inspiratory effort sensation threshold to added inspiratory resistive loading that was reversed following CPAP therapy. In earlier work, OSA patients revealed an elevated threshold in the detection of flow‐resistive load (McNicholas et al., 1984), and a reduced ventilatory load compensation (Greenberg & Scharf, 1993). However, the way effort perception is assessed influences the outcomes generated and previous work did not differentiate between the perceived effort during resistive breathing and the effort sensitivity alterations (i.e., force/pressure changes) seen after repetitive loading.

Possible effects of consecutive loading cycles of the respiratory system on effort perception (i.e., sensitivity) have also not been investigated, as well as its response to CPAP treatment. Moreover, the inclusion of muscular parameters (pressure/force and electromyography [EMG]) for establishing fatigue responses of respiratory and accessory muscles in OSA patients is lacking. Consequently, this study uses repetitive loading cycles of the inspiratory muscles for assessing the effort perception between loading cycles with additional measures of inspiratory pressure and EMG of inspiratory (intercostal) and accessory (trapezius) muscles in newly diagnosed OSA patients and healthy controls. In addition, an analogous loading protocol is applied to leg muscles (quadriceps) with measures of forces plus EMG for the investigation of potentially generalized effects of OSA on effort perception. Protocols were repeated after 3‐months of CPAP treatment to investigate potential adaptation of effort perception. In addition, healthy controls were separated into high and low fitness groups to investigate cardiovascular fitness as a potential factor for alterations seen in OSA patients. We hypothesized that repetitive inspiratory and limb muscle loading would lead to a stronger elevation of muscular fatigue parameters in OSA patients compared with healthy controls. In addition, we hypothesized that OSA patients would reveal a stronger alteration of effort perception during repetitive loading cycles compared with healthy participants and that CPAP treatment ameliorates detected effects. In addition, we expected that levels of effort sensitivity would be associated with OSA severity that is, AHI.

## 2 METHODS

### 2.1 Participants

**FIGURE 1:**
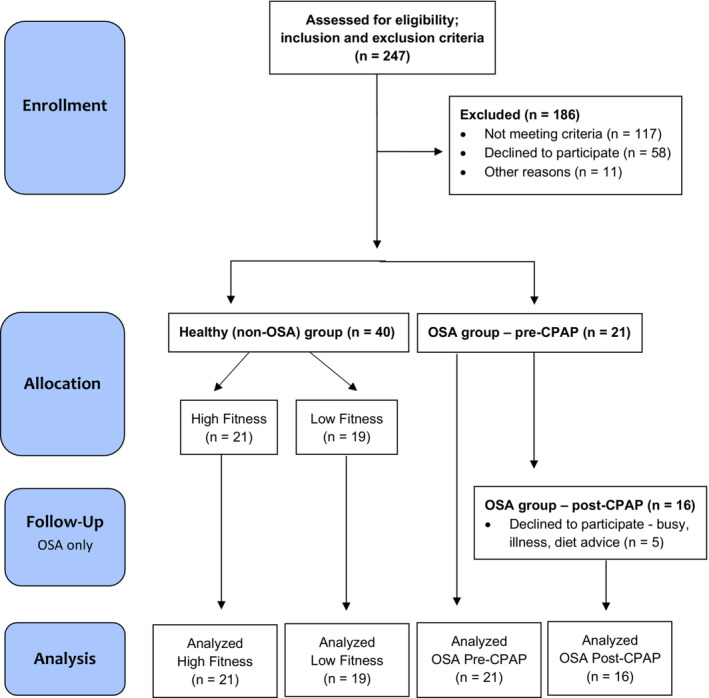
Flow diagram of the study design (adapted from CONSORT guidelines template).

The study was approved by research ethics committees at the School of Human and Behavioural Sciences, Bangor University (P01‐16/17) and National Health Service (NHS) ethics committee East Midlands—Leicester South, Nottingham (17/EM/0162), conforming to the Declaration of Helsinki. Written informed consent was obtained from all participants before enrolling in the study. Data were anonymised after collection and individuals could not be identified by authors. For the combined cross‐sectional and cohort study (Figure 1), newly diagnosed OSA male patients were enrolled in the Pulmonary Function Department (Ysbyty Gwynedd, Bangor) between July 2017 and January 2019. An overnight home‐based sleep study was completed patients to determine the AHI of all patients as per routine (Konica Minolta; Nox Medical). The diagnostic process was performed by registered clinical physiologists according to the criteria of the American Academy of Sleep Medicine Task Force (Berry et al., 2012).

Newly diagnosed patients with OSA were deemed not eligible for study participation if they were aged >70 years old, with a body mass index (BMI) ≥ 40 kg/m^2^, taking pharmacological treatment (e.g., opioid‐analgesics), reporting musculoskeletal/orthopedic injuries, and history of chronic disease. OSA patients were reassessed after 3 months of CPAP treatment as per departmental guidelines (AirSense™, ResMed Limited). Healthy male participants were recruited from the general population of North Wales, UK, aged between 18 and 45 years old from August 2016 to September 2017. Exclusion criteria were a BMI > 39.9 kg/m^2^, taking pharmacological treatment (e.g., opioid‐analgesics), and reporting musculoskeletal/orthopedic injury, medical condition, and/or sleep disorder according to the Epworth Sleepiness Scale (ESS >10) and Pittsburgh Sleep Quality Index (PSQI >5) (Buysse Charles et al., 1989; Johns, 1991).

### 2.2 Overall study design

The study examined the impact of inspiratory and limb muscle loading on effort perception and muscle activity via surface EMG in OSA patients and healthy individuals. All participants were required to visit the laboratories on three separate occasions with at least 72 h between visits. During the first visit, participants' body composition and pulmonary function were recorded. In healthy (non‐OSA) individuals, an incremental exercise protocol was then performed to determine cardiorespiratory fitness. During the second and third visits, all participants completed the inspiratory and limb muscle loading protocols assessing effort perception, muscular forces, and inspiratory pressures, as well as muscular activity via surface EMG. An additional visit was scheduled for OSA patients after receiving 3 months of CPAP treatment to assess body composition, pulmonary function, and repeat the inspiratory loading protocol.

## 3 MEASUREMENTS

### 3.1 Participants' characteristics

Participants' height and weight were measured using a stadiometer and digital platform scale (SECA). Spirometry measures consisting of forced expiratory volume in 1 s (FEV_1_), forced vital capacity (FVC), and FEV_1_/FVC ratio were assessed in line with established guidelines (MicroMedical Ltd) (Miller et al., 2005). The diffusion capacity for carbon monoxide (DLCO) and total lung capacity (TLC) of OSA patients were assessed in line with standardized procedures (Viasys VMAX Encore, SensorMedics Corporation) (Macintyre et al., 2005). Healthy participants performed an incremental exercise test on a cycle ergometer to determine cardiorespiratory fitness (Lode Excalibur Sport) (ACSM, 2003; Murias et al., 2018). Participants were instructed to cycle at 60–90 rpm at 50 W, with increments of 20 W occurring every 1 min until volitional exhaustion. Heart rate and rate of perceived exertion (RPE) were recorded during the final 15 s of each 1‐min stage using a heart rate monitor and Borg 6–20 scale (Polar Electro Oy) (Borg, 1998). Expired gases were measured breath‐by‐breath using a metabolic analyzer (CORTEX Biophysik GmbH, Germany) with the maximal oxygen uptake (VO_2max_) defined as the highest 30 s average of oxygen uptake (VO_2_). The criteria for VO_2max_ were also achieved if two of the following occurred: plateau in VO_2_ despite the increasing workload, heart rate within 10 beats of predicted maximum heart rate, respiratory exchange ratio ≥1.15, and cadence <60 rpm despite strong verbal encouragement (Edvardsen et al., 2014; Howley et al., 1995; Midgley et al., 2007). Participants were then stratified into either High fitness (VO_2max_ > 55 mL/kg·min) or low fitness (VO_2max_ < 40 mL/kg·min) groups according to their cardiorespiratory fitness (Astorino et al., 2012; Hassmén, 1990). Any participant with a VO_2max_ that fell between the two classified groups was excluded from any further study participation. The high fitness group was selected based on the possible adaptations elicited from endurance training to both sets of muscles and control of effort sensitivity during loading. In contrast, low fitness group was recruited to ensure individuals would experience muscular fatigue associated more so with sedentary behavior, but no hypoxic stress as is typically observed in OSA.The safety consideration in OSA patients (i.e., larger BMI and cardiac stress) and no supervision/oversight from other health professionals meant that maximal exercise testing was not performed in this higher risk population.

### 3.2 Electromyography (EMG)

Intercostal and trapezius EMG signals were acquired with bipolar pairs of silver/silver chloride electrodes placed on cleaned and abraded skin of the fifth intercostal space (posterior axillary line) for the intercostal muscle and between the spinous C7 process and acromion process for the upper trapezius muscle (Ambu® Neuroline 720–45 × 22 mm, Denmark; MP150 system, BIOPAC Systems Inc) (Cescon et al., 2008; Hawkes et al., 2007). The ground electrodes were placed on the sternum and C7 process. All electrodes were positioned on the right side of the body to minimize interference from electrocardiogram currents and attempts were made to align electrodes with the orientation of muscle fibers. The interelectrode distance was minimized to <2 cm. A clear EMG signal was obtained by asking participants to take a deep inspiration and shoulder shrug before securing cables with surgical tape to reduce motion artifacts. Many studies investigating the intercostal EMG activity during loaded and unloaded breathing have selected the external intercostal muscles within the fifth intercostal space at the posterior axillary line (De Troyer et al., 1998; Hawkes et al., 2007; Hershenson et al., 1989). While the upper trapezius muscle is a lesser‐known accessory muscle of inspiration it is crucial for supporting and stabilizing the ribcage during loaded breathing (Tokizane et al., 1952; Yokoba et al., 2003). Vastus medialis EMG signals of the dominant leg were acquired with bipolar pairs of silver/silver chloride electrodes placed longitudinally on shaved, cleaned, and abraded skin in accordance with SENIAM guidelines (Ambu® Neuroline 720–45 × 22 mm; Powerlab‐16 SP, ADInstruments) (Hermens et al., 2000). A ground electrode was placed on the superior aspect of the patella.

### 3.3 Inspiratory muscle loading protocol

The experimental loading protocol and rating of perceived exertion (RPE 14—somewhat hard/hard) assessment were performed according to Earing et al., 2014 (2014) and designed to match the limb muscle loading protocol reported below. The test–retest reliability of the inspiratory loading and RPE protocol has been assessed in a former study with 37 healthy participants (BMI: 23.1 ± 1.5 kg/m^2^) and 26 overweight participants (BMI: 28.2 ± 2.5 kg/m^2^). Test–retest intraclass correlation coefficients were assessed for the inspiratory pressures generated at RPE14 after each set of 20 loaded breaths at 50% maximal inspiratory pressure (IP_max_) using the Powerbreathe Plus device (POWERbreathe International Ltd). A second assessment was conducted after 1 week under identical testing conditions. The protocol implemented by Earing et al., (2014) revealed an excellent test–retest reliability of 0.967 with a 95% confidence interval (CI) of 0.953–0.978.

**FIGURE 2:**
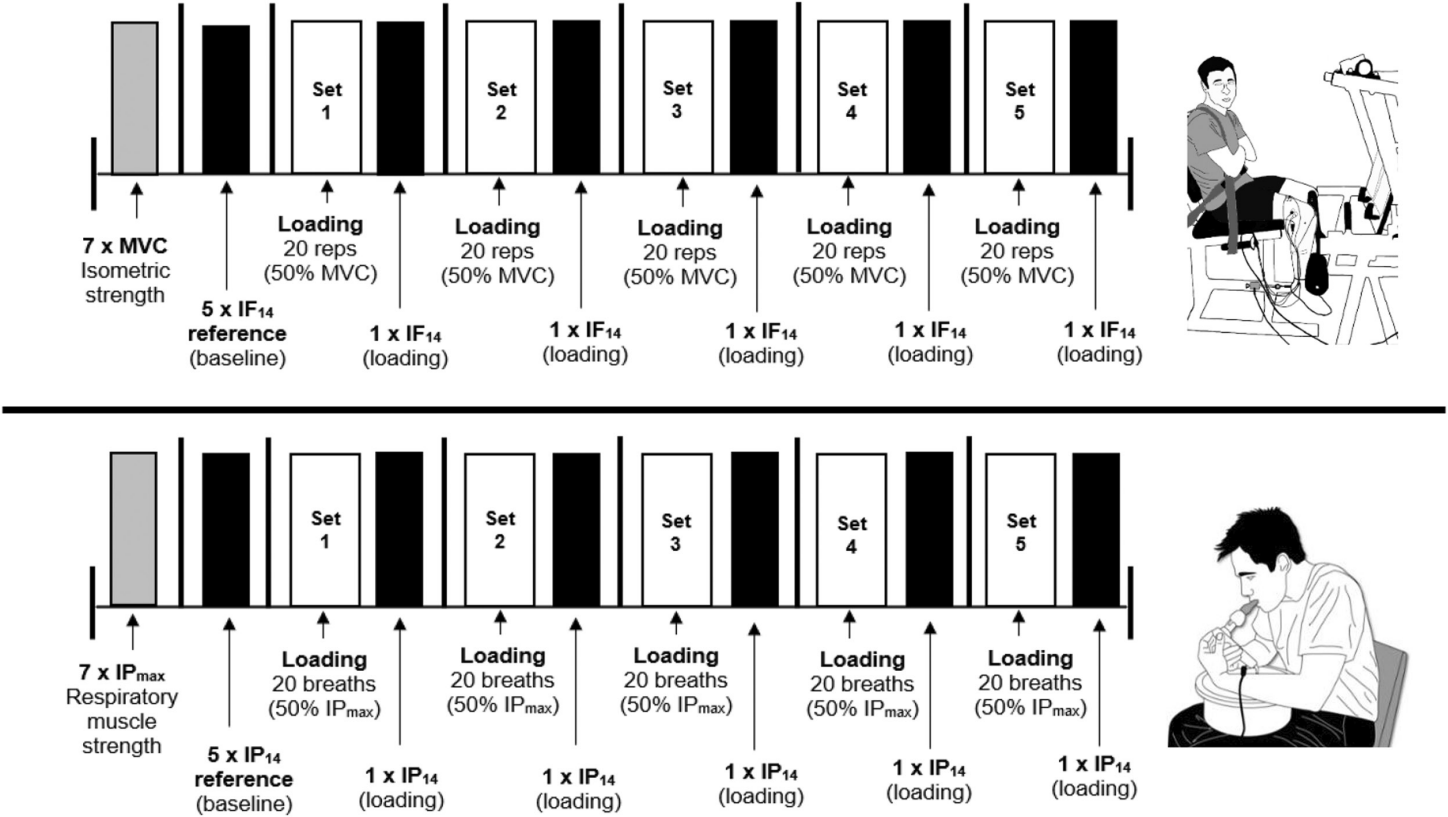
Schematic diagram of the limb and inspiratory muscle loading protocols. IF_14_, Isometric force at RPE14; IP_14_, Inspiratory pressure at RPE14; IP_max_, Maximal Inspiratory Pressure; MVC, maximal voluntary contraction.

The current study utilized the detailed methodology as described below. The IP_max_ was measured from residual volume using the Powerbreathe KH2 electronic inspiratory‐muscle training device to determine participants' respiratory muscle strength and set the pressure of the loading protocol (POWERbreathe International Ltd) (Figure 2). Each participant was instructed to produce seven consecutive maximal efforts for 3 s while receiving strong verbal encouragement and visual feedback on a computer screen. A rest interval of 1 min separated each maximal effort. The highest pressure generated from the best trial was defined as the IP_max_ (cmH_2_O).

The loading protocol was then performed after 5 min of rest. Participants were instructed to generate five inspiratory pressures at a constant effort of RPE14 which corresponded to the verbal anchors of “somewhat hard” to “hard/heavy” on the 6–20 Borg RPE scale (Borg, 1998) for 3 s without visual feedback. The inspiratory pressure, generated by inspiratory muscle activation, at a distinct effort scaling point can be understood as a measure of effort sensitivity. Higher muscle recruitment at the same level of perceived effort would generate higher inspiratory pressure, and therefore would constitute a lower effort sensitivity versus a reduced inspiratory pressure at the same effort level would constitute an elevated effort sensitivity. A rest interval of 30 s separated each inspiratory pressure generated. The inspiratory loading protocol was performed consisting of five sets of 20 loaded breaths at a resistance equivalent to 50% IP_max_. Participants were instructed to breathe naturally (not accelerated) during the loading protocol. The target force (50% IP_max_) was set up on a monitor in full view of participants. After each set of loaded breaths, participants were instructed to produce the inspiratory pressure at RPE14 for 3 s without visual feedback.

### 3.4 Limb muscle loading protocol

A custom‐made isometric chair with an immovable pad located proximal to the ankle joint at a 90° angle was used to load the quadriceps muscles of participants (Model 615, Vishay Tedea‐Huntleigh; ML110 Bridge Amplifier, Powerlab‐16 SP, ADInstruments). After familiarization and warm‐up, the maximal voluntary contraction (MVC) was measured to determine participants' quadriceps muscle strength and adjust the force for the loading protocol (Figure 2). Participants were instructed to produce seven consecutive maximal efforts with the dominant leg for 3 s while receiving strong verbal encouragement and visual feedback on a computer screen. A rest interval of 1 minute separated each maximal effort. The maximal force generated from the best trial was defined as the MVC.

**FIGURE 3:**
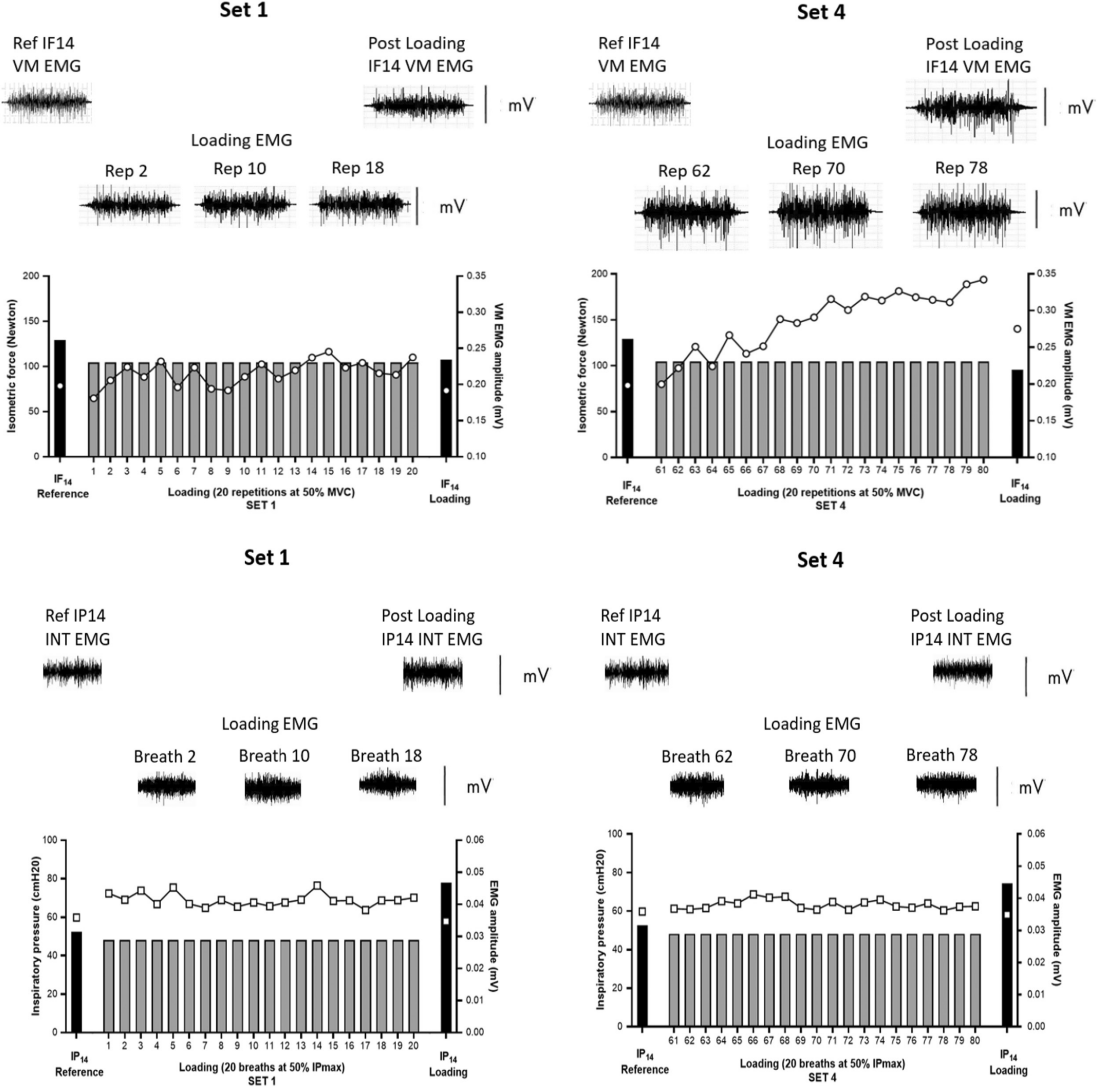
Schematic diagram illustrating the individual limb and inspiratory muscle response to loading (20 and 80 repetitions/breaths) in OSA patients. EMG, Electromyography; IF_14_, Isometric force at RPE14; INT, Intercostal; IP_14_, Inspiratory pressure at RPE14; IP_max_, Maximal Inspiratory Pressure; MVC, Maximal Voluntary Contraction; VM, Vastus Medialis.

After 5 min of rest, participants were instructed to generate five isometric forces at a constant effort of RPE14. Each isometric force was held for 3 s and separated by 30 s of rest. No visual feedback was provided during these measures to ensure participants focused entirely on their perceived effort as a gauge. This was followed by the limb muscle loading protocol which consisted of five sets of 20 isometric contractions at 50% MVC. Each contraction was held for 3 s followed by a rest interval of 3 s and repeated continually until complete. The target force (50% MVC) during the loading was set up on a monitor in full view of participants. After each set of isometric contractions, participants were instructed to generate an isometric force at RPE14 for 3 s without visual feedback. An example of limb and inspiratory muscles' responses to the loading protocol in an OSA patient (EMG and force/pressure) is shown in Figure 3.

### 3.5 Data analysis

Inspiratory pressures generated at RPE14 were studied according to the absolute pressure (cmH_2_O) and relative values (normalized according to IP_max_ and expressed as % IP_max_). The series of inspiratory pressures generated at RPE14 before and after loading were also averaged and referred to as IP_14_ baseline and IP_14_ loading. Isometric forces generated at RPE14 were studied according to absolute forces (N) and relative values (normalized according to MVC and expressed as % MVC). The series of isometric forces generated at RPE14 before and after loading were averaged and referred to as the IF_14_ baseline and IF_14_ loading. EMG signals were amplified (gain x 1000), band‐pass filtered between 35 and 500 Hz, digitized at a sampling rate of 2000 kHz, and later analyzed offline using either Power Lab Chart 4.2.3 or AcqKnowledge 3.9 software (ADInstruments, Australia; BIOPAC Systems Inc., US) (Deschenes et al., 2008; Roussos et al., 1979). Root mean square (RMS) amplitudes with a time constant of 100 ms were analyzed over a 0.5 s window (the point at which maximal pressure/force was generated). Data were reported as amplitude (mV) and normalized according to the maximal EMG (% RMS).

### 3.6 Statistical analysis

The sample size was calculated using G*Power software (version 3.1.2) with 80% power at a 5% significance level based on former cross‐sectional study data examining the inspiratory effort perception after loaded breathing in 26 healthy overweight participants and 24 OSA patients revealing an effect size of 0.49 (Earing, 2014). The present study, therefore, aimed to recruit a total of 40 healthy participants and 20 OSA patients to detect a moderate effect size and account for the potential drop‐out of participants. Data were expressed as mean ± SD unless otherwise stated. Statistical significance was considered as *p* < 0.05. All variables of interest were tested for parametric test assumptions. The group's characteristics were compared using one‐way ANOVA. The slopes for limb and inspiratory muscles' EMG amplitudes during loading were assessed by linear regression analyses. The relative force/pressure generated at RPE14 and percentage change, the relative EMG amplitudes for inspiratory and limb muscles, were analyzed using one‐way ANOVA or paired *t‐*tests. Bonferroni tests were performed for all post hoc analyses. The effect of CPAP treatment on body characteristics, lung function, pressures generated at RPE14, and percentage change were analyzed using paired *t‐*tests. Spearman's rho examined potential correlations between patients' AHI and IP_14_ at baseline and following loading. Statistical analyses were performed using SPSS version 25 for Windows (SPSS Inc.).

## 4 RESULTS

### 4.1 Participant characteristics

Newly diagnosed patients with OSA had a significantly higher BMI (obese category) and were older than healthy controls (Table 1). The AHI of OSA patients was in the severe category (AHI ≥30) and sleep‐related questionnaires (ESS and PSQI) were significantly higher in OSA patients compared with healthy individuals reporting normal scores. Pulmonary function (FEV_1_/FVC ratio) was significantly lower in OSA patients, however, in the range of age, height, and weight‐corrected predictions (Roca et al., 1998). No significant differences were found between the groups' maximal inspiratory muscle strength (IP_max_). However, the maximal strength of the quadriceps muscles (MVC) was found to be ~30% lower in OSA patients compared with healthy individuals.

**TABLE 1:** Participant characteristics.

	High fitness (*n* = 21 males)	Low fitness (*n* = 19 males)	OSA (*n* = 21 males)
Age (years)	35.1 (9.6)	27.8 (7.8)^b^	55.9 (9.1)^d^
Height (cm)	179.5 (6.8)	176.1 (8.6)	173.4 (6.0)^c^
Weight (kg)	76.5 (11.0)	79.7 (15.6)^b^	101.5 (19.9)^d^
BMI (kg/m^2^)	23.7 (3.0)	25.6 (3.60)^b^	33.7 (6.1)^d^
AHI (events/h)	ND	ND	42.8 (27.2)
ESS	5.4 (4.1)	2.9 (2.0)^b^	11.5 (5.6)^d^
PSQI	3.4 (2.0)	4.2 (2.1)^b^	8.6 (4.1)^d^
FEV_1_ (L/min)	4.48 (0.7)	4.17 (0.5)^b^	3.20 (0.49)^d^
FVC (L/min)	5.44 (0.7)	5.04 (0.6)^b^	4.16 (0.78)^d^
FEV_1_/FVC (%)	82.4 (6.4)	83.3 (8.4)^a^	76.6 (6.2)^c^
VO_2max_ (ml/kg·min)	61.0 (8.4)	39.7 (5.8)	ND
IP_max_ (cmH_2_O)	111.3 (21.8)	112.7 (27.4)	104.0 (19.5)
MVC (N)	411.7 (97.7)	382.4 (120.1)	280.8 (114.3)^d^

*Note*: Values shown are mean ± SD. Data were analyzed using one‐way ANOVA.

Abbreviation: AHI, apnoea hypopnea index; BMI, Body mass index; ESS, Epworth Sleepiness Scale; PSQI, Pittsburgh sleep quality index; FEV_1_, forced expiratory volume 1 s; FVC, Forced vital capacity; VO_2max_, maximal oxygen uptake; IP_max_, maximal inspiratory pressure; MVC, Maximal voluntary capacity; ND, Not determined.

^a^ *p* < 0.05;

^b^ *p* < 0.01 OSA vs Low Fitness;

^c^ *p* < 0.05;

^d^ *p* < 0.01 OSA vs High Fitness.

The compliance of OSA patients receiving CPAP treatment was good over the course of 3 months (mean pressure: 8.67 ± 2.9 cmH_2_O, usage: 7.06 ± 1.4 h/night [4.11–9.23 h/night], and days of usage >4 h: 93.7 ± 8.9%). CPAP treatment produced significant reductions in patients' AHI, ESS, and PSQI scores (Table 2). No alterations in pulmonary function and maximal inspiratory muscle strength (IP_max_) were detected after CPAP treatment.

**TABLE 2:** Effect of CPAP treatment on participant characteristics.

	Pre‐CPAP (*n* = 15 males)	Post‐CPAP (*n* = 15 males)
Age (years)	55.2 (9.3)	ND
Height (cm)	173.3 (5.3)	173.3 (5.3)
Weight (kg)	99.7 (22.3)	101.6 (22.4)^b^
BMI (kg/m^2^)	33.1 (6.7)	33.7 (6.7)^b^
AHI (events/h)	39.2 (23.2)	4.4 (3.4)^b^
ESS	11.5 (4.9)	4.3 (2.6)^b^
PSQI	9.4 (3.6)	4.5 (2.5)^b^
FEV_1_ (L/min)	3.26 (0.40)	3.26 (0.33)
FVC (L/min)	4.29 (0.62)	4.25 (0.52)
FEV_1_/FVC (%)	76.4 (5.9)	76.5 (5.8)
DLCO (ml/min/mmHg)	23.2 (4.7)	24.5 (5.0)
TLC (L/min)	6.14 (0.89)	6.75 (0.90)^a^
IP_max_ (cmH_2_O)	104.6 (16.8)	105.7 (17.1)

*Note*: Values shown are mean ± SD. Data were analyzed using paired *t*‐tests.

Abbreviations: AHI, apnea hypopnea index; BMI, Body mass index; DLCO, diffusing capacity of the lung for carbon monoxide; ESS, Epworth sleepiness scale; FEV_1_, forced expiratory volume 1 s; FVC, Forced vital capacity; IP_max_, maximal inspiratory pressure; ND, not determined; PSQI, Pittsburgh Sleep Quality Index; TLC, total lung capacity.

^a^ *p* < 0.05;

^b^ *p* < 0.01 Pre vs Post‐CPAP.

## 5 LOADING PARADIGM

### 5.1 Limb and inspiratory muscle electromyography (EMG) during loading

**FIGURE 4:**
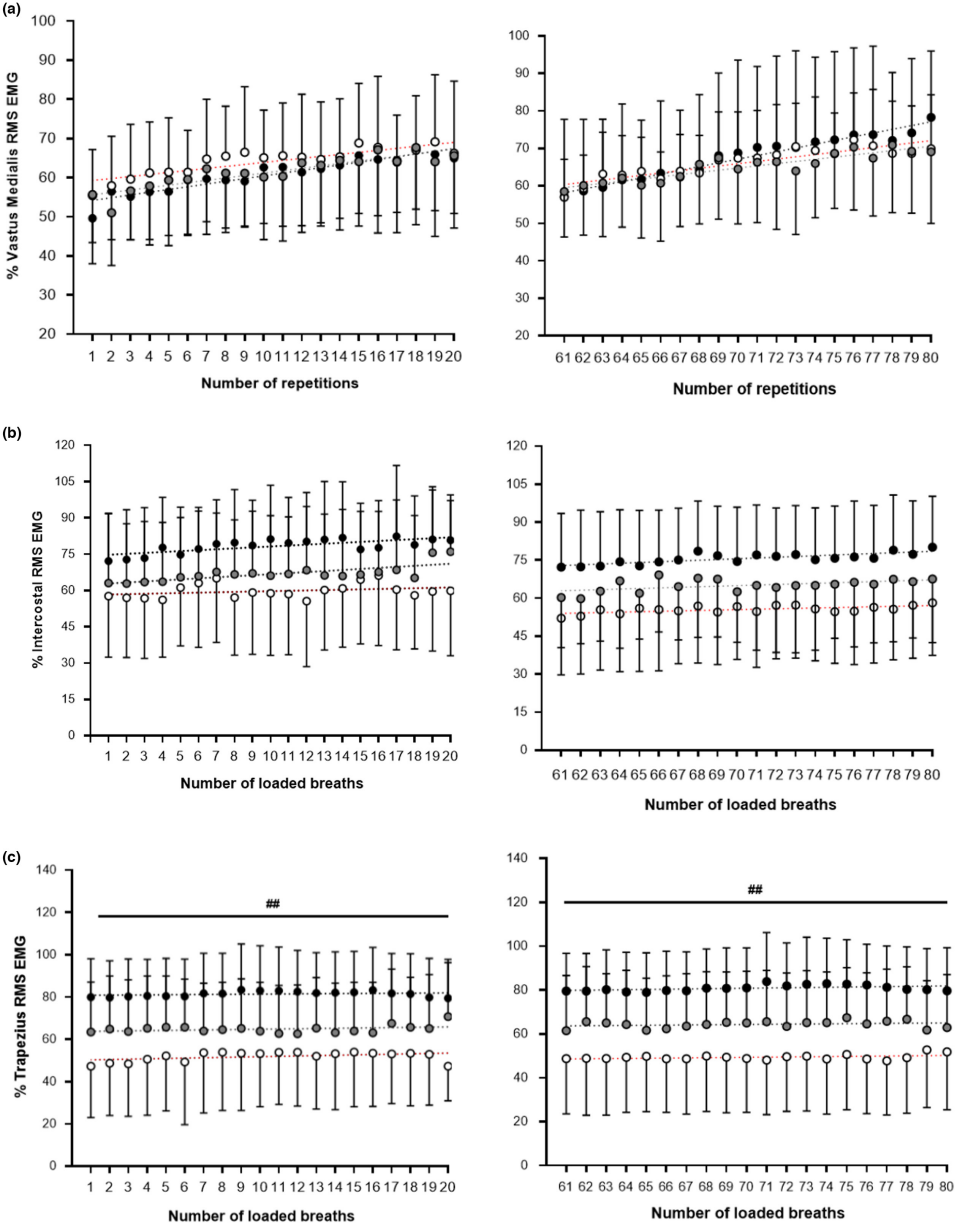
Limb (A) and inspiratory electromyography (B,C) (EMG) during loading. EMG data is expressed as Mean ± SD. Data was analyzed using One‐Way ANOVA. (white circles) = High fitness group (*n* = 18); (gray circles) = Low fitness group (*n* = 17); (black circles) = OSA group (*n* = 14). The dashed line represents the linear trend line. EMG, electromyography; RMS, root mean square. ^##^ *p* < 0.01 versus high fitness group.

The loading protocols for the limb and inspiratory muscles produced signs of fatigue as reflected by the change in EMG amplitudes (Figure 4). The vastus medialis EMG amplitude increased during loading and increased between sets in both OSA patients (first set: *B* = 0.696 μV/set, *p* = 0.000; 4th set: *B* = 0.997 μV/set, *p* = 0.000) and High fitness individuals (first set: *B* = 0.513 μV/set, *p* = 0.000; fourth set: *B* = 0.616 μV/set, *p* = 0.000) (Figure 4a). Whereas the Low fitness group demonstrated increases during loading (first set: *B* = 0.616 μV/set, *p* = 0.000; fourth set: *B* = 0.582 μV/set, *p* = 0.000). The data in full are shown in Table 3.

**TABLE 3:** Slopes of limb and inspiratory muscle EMG amplitudes during loading.

		First set of loading (20 isometric loading/loaded breaths)					Fourth set of loading (80 isometric loading/loaded breaths)				
	Group	*B*	*SE B*	β	*t*	*p*	*B*	*SE B*	β	*t*	*p*
VM (μV/set)	High fitness	0.513	0.077	0.845	6.693	0.000	0.616	0.072	0.895	8.523	0.000
	Low fitness	0.616	0.074	0.891	8.341	0.000	0.582	0.061	0.914	9.531	0.000
	OSA	0.696	0.065	0.930	10.742	0.000	0.997	0.054	0.975	18.595	0.000
INT (μV/set)	High fitness	0.152	0.114	0.300	1.333	0.199	0.165	0.047	0.634	3.477	0.003
	Low fitness	0.431	0.093	0.738	4.639	0.000	0.229	0.086	0.531	2.656	0.016
	OSA	0.380	0.082	0.737	4.627	0.000	0.296	0.055	0.787	5.413	0.000
TRA (μV/set)	High fitness	0.169	0.085	0.424	1.986	0.062	0.084	0.044	0.412	1.916	0.071
	Low fitness	0.110	0.070	0.349	1.581	0.131	0.071	0.064	0.253	1.111	0.281
	OSA	0.056	0.049	0.261	1.145	0.267	0.109	0.051	0.452	2.147	0.046

*Note*: The coefficients are shown per one‐unit change for each independent variable. A positive coefficient reflects an increase in the outcome variable per unit of the predictor variable (e.g., VM EMG increases by 0.045 μV from the first to fourth set of loading in OSA patients).

Abbreviations: VM, Vastus Medialis; INT, Intercostal; TRA, Trapezius.

Repetitive loading of the inspiratory muscles led to an increased EMG amplitudes of the intercostal muscles confirming effectiveness of the loading protocol. Newly‐diagnosed OSA patients (first set: *B* = 0.380 μV/set, *p* = 0.000; 4th set: *B* = 0.296 μV/set, *p* = 0.000) and low fitness individuals (first set: *B* = 0.431 μV/set, *p* = 0.000; fourth set: *B* = 0.229 μV/set, *p* = 0.016; Figure 4b) generated higher Intercostal EMG amplitude increases during loading. In contrast, High fitness individuals demonstrated only slight increases in Intercostal EMG amplitudes between sets (first set: *B* = 0.152 μV/set, *p* = 0.199; 4th set: *B* = 0.165 μV/set, *p* = 0.003). The trapezius EMG amplitudes increased only marginally during the fourth set of loading in OSA patients (first set: *B* = 0.056 μV/set, *p* = 0.267; 4th set: *B* = 0.109 μV/set, *p* = 0.046; Figure 4c). However, results demonstrated that the OSA patients recruited the trapezius muscle to a significantly greater extent during loading in comparison with High fitness individuals. The relative trapezius EMG amplitude (% RMS) was significantly higher during loading in OSA patients than in high fitness individuals (first set: *p* = 0.003; fourth set: *p* = 0.001).

### 5.2 Isometric force and inspiratory pressure measured at RPE14

**FIGURE 5:**
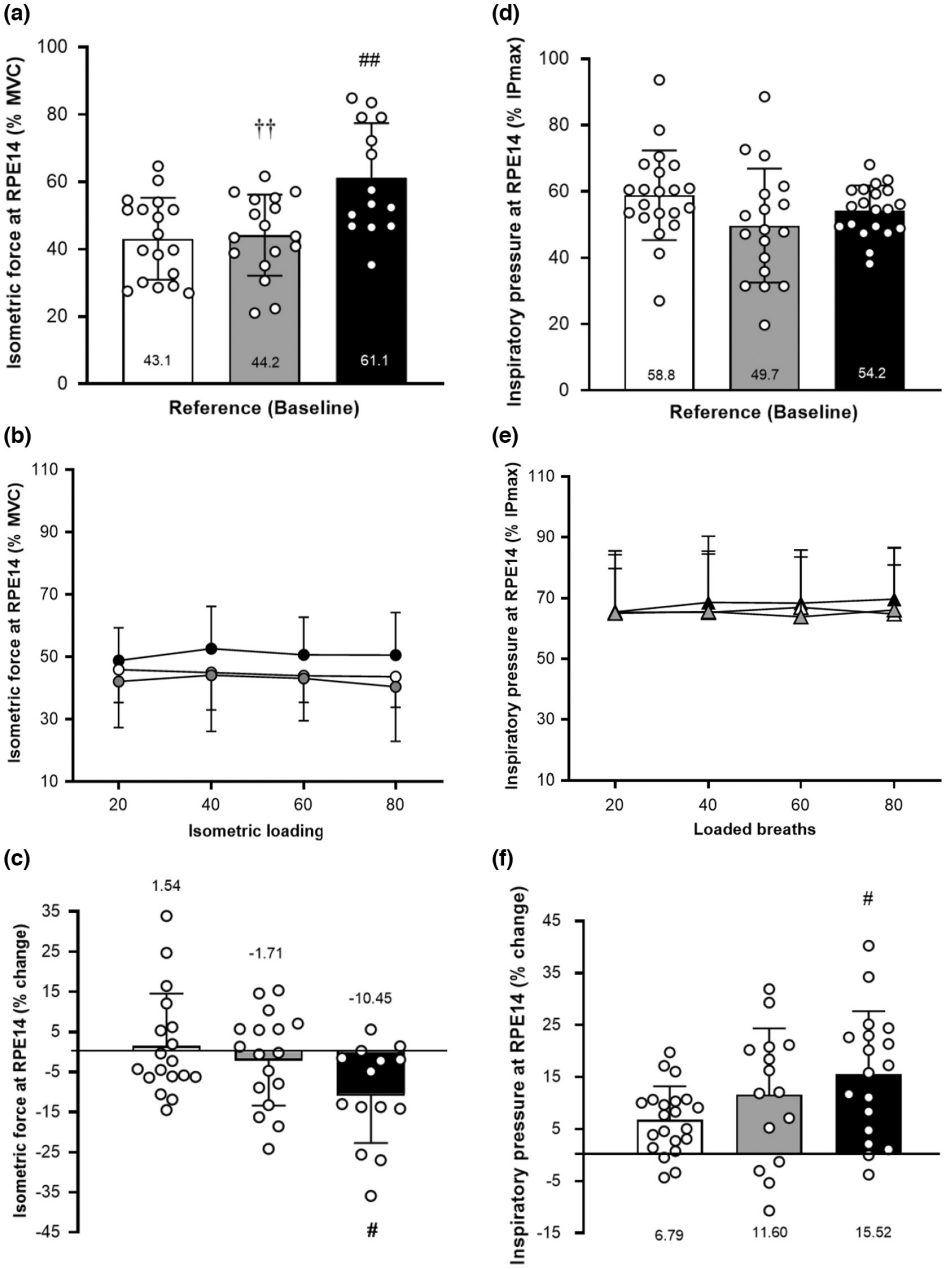
Isometric forces and inspiratory pressures generated at RPE14: baseline (A,D), after loading (B,E), and percentage change (C,F). Force/pressure data are expressed as mean ± SD. Data were analyzed using one‐way ANOVA. Limb data: (white bars/circles) = high fitness group (*n* = 18); (gray bars/circles) = low fitness group (*n =* 17); (black bars/circles) = OSA group (*n* = 14). Inspiratory data: (white bars/triangles) = High fitness group (*n* = 21); (gray bars/triangles) = Low fitness group (*n =* 17); (black bars/triangles) = OSA group (*n* = 18). IP_max_, maximal inspiratory pressure; MVC, maximal voluntary contraction. ^††^ *p* < 0.01 versus OSA group. ^#^ *p <* 0.05 and ^##^ *p* < 0.01 versus high fitness group.

The isometric limb forces and inspiratory pressures generated at a perceived effort of 14 (somewhat hard–hard) at baseline and following loading are shown in Figure 5. For the limb muscles, the relative isometric forces produced at RPE14 (IF_14_) were significantly different between the groups at baseline (*p* = 0.001). Patients with OSA generated significantly higher relative IF_14_ at baseline when compared with both high fitness (*p* = 0.001) and low fitness individuals (*p* = 0.003; Figure 5a). Consequently, OSA patients used a higher proportion of their muscular capacity at a fixed RPE of 14 than healthy individuals. Further analyses were performed to examine the impact of limb muscle loading on forces produced at RPE14 (IF_14_). No significant between‐group differences in the relative IF_14_ were observed following loading and no alterations were detected between sets (Figure 5b). We further examined the difference in IF_14_ between baseline and loading, with statistical analyses revealing that loading influenced IF_14_ significantly (*p* = 0.028, Figure 5c). The percentage change between baseline and loading IF_14_ was significantly larger in OSA patients than in high fitness individuals (*p* = 0.027). OSA patients generated lower forces at RPE14 after loading unlike the responses of healthy individuals who produced forces matching their IF_14_ at baseline after loading.

For the inspiratory muscles, a contrasting set of results were observed compared with the limb muscle outcomes. At baseline, all groups produced a similar percentage of their IP_max_ at RPE14 (IP_14_ at ~50%–60% of IP_max_) (Figure 5d). However, unlike the limb muscles, loading induced significant increases in pressure generated at an RPE of 14 in all groups (*p* = 0.012). Indeed, OSA patients responded to loading with a significantly larger percentage increase of IP_14_ compared with high fitness individuals (*p* = 0.037, Figure 5f). Moreover, this effect was mainly induced by the first loading cycle; within the repeated bouts of loading, no further changes in IP_14_ were detected in all groups (Figure 5e). Consequently, the first loading cycle induced a strong increase of pressure generated at an RPE of 14 in the respiratory system with OSA patients showing the strongest response to loaded breathing. This change in effort sensitivity was specific to the respiratory system and not detected following limb muscle loading.

### 5.3 Electromyography (EMG) of limb and inspiratory muscles at RPE14

**FIGURE 6:**
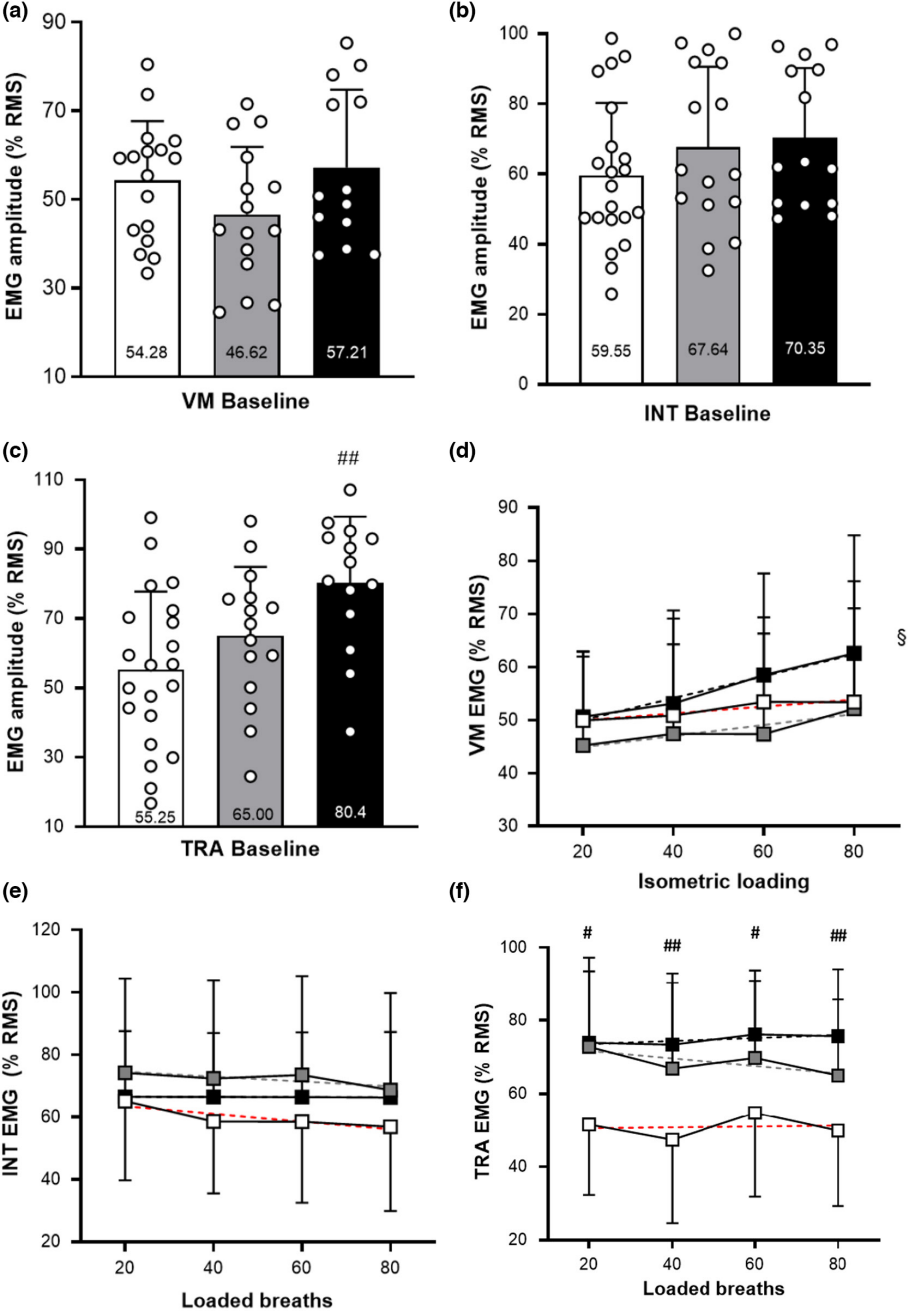
Limb and inspiratory EMG amplitude measured at RPE14: baseline (A–C) and after loading (D, E, F). EMG data are expressed as mean ± SD. Data were analyzed using One‐Way ANOVA. VM data: (white bars/squares) = high fitness group (*n* = 18); (gray bars/squares) = low fitness group (*n =* 17); (black bars/squares) = OSA group (*n* = 14). INT/TRA data: (white bars/squares) = high fitness group (*n* = 21); (gray bars/squares) = low fitness group (*n =* 16); (black bars/squares) = OSA group (*n* = 14). ^#^ *p* < 0.05, ^##^ *p* < 0.01 versus High fitness group. The dashed line represents the linear trend line. ^§^ *p* < 0.01, Slope of Vastus Medialis muscle activity in OSA patients analyzed using linear regression analysis.

Limb and inspiratory EMG amplitudes measured during IF_14_ and IP_14_ respectively at baseline and following loading are presented in Figure 6. For the limb muscles, no significant between‐group differences were found in the relative EMG amplitudes (% RMS) of the Vastus Medialis muscle at baseline and after loading (*p* > 0.05; Figure 6a,d). However, the relative EMG amplitudes for the vastus medialis muscle at RPE14 increased in response to loading in the OSA group. Linear regression analysis revealed an increased slope of vastus medialis muscle recruitment at RPE14 following loading in OSA patients (*B*: 0.206 μV/N, *p* = 0.009). In contrast, healthy individuals maintained the vastus medialis EMG amplitude without any increase over the loading cycles (*p* > 0.05; Figure 6d).

For the intercostal muscles, there were no significant between‐group differences in the relative IP_14−_EMG amplitudes (% RMS) at baseline and following loading (*p* > 0.05; Figure 6b,e). However, the relative trapezius IP_14_–EMG amplitude was found to be significantly larger at baseline in OSA patients than in high fitness individuals (*p* = 0.003). Furthermore, the relative EMG amplitudes of the trapezius muscles (% RMS) at RPE14 were different between groups following loading (*p* = 0.003), with post‐hoc analyses indicating that OSA patients demonstrated a significantly higher IP_14_–EMG amplitude compared with high fitness individuals (all *p* < 0.05; Figure 6f). Furthermore, the higher recruitment of Trapezius muscles was observed during all IP_14_ measures in OSA and low fitness groups (Figure 6f).

### 5.4 Effect of CPAP treatment on inspiratory pressures generated at RPE14

**FIGURE 7:**
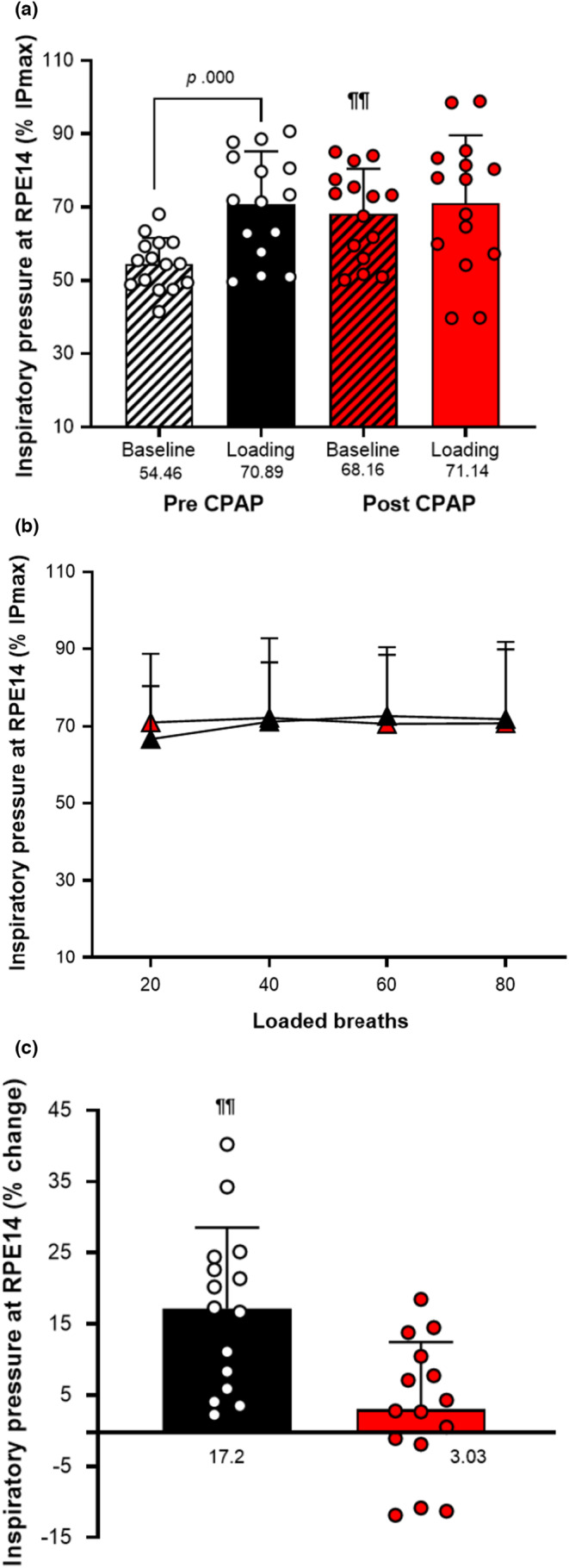
Effect of CPAP treatment on inspiratory pressures generated at RPE14: baseline (a), after loading (b), and percentage change (c). Pressure data are expressed as Mean ± SD. (black bars/triangles) = pre‐CPAP group (*n* = 15); (red bars/triangles) = Post‐CPAP group (*n* = 15). IP_max_, Maximal Inspiratory Pressure. Significant differences reported Post‐CPAP as shown by ^¶¶^ *p* < 0.01 analyzed using paired *t*‐tests.

Fifteen patients with OSA were retested with the inspiratory muscle loading paradigm after receiving 3‐months of CPAP treatment. Following CPAP therapy, OSA patients generated significantly higher absolute and relative IP_14_ values at baseline (*p* = 0.000; Table 4 and Figure 7a) compared with pre‐CPAP. After loading, patients generated identical levels of IP_14_ as before CPAP (*p* > 0.05, Figure 7b), revealing that CPAP treatment only influenced the IP_14_ generated before loading (baseline measure). Consequently, the alterations in IP_14_ following loading in OSA patients dissipated after receiving CPAP treatment for 3 months. The percentage change in IP_14_ between baseline and loading was significantly reduced after CPAP treatment (*p* = 0.000, Figure 7c).

**TABLE 4:** Effect of CPAP treatment on inspiratory pressures generated at RPE14.

	Inspiratory muscle data (cmH_2_O)	
	Pre‐CPAP (*n* = 15)	Post‐CPAP (*n* = 15)
IP_max_	104.6 (16.8)	105.7 (17.1)
IP_14_ baseline	56.7 (10.2)	77.0 (13.8)^c^
IP_14_ 20 breaths	68.7 (11.7)^a^	74.6 (21.8)
IP_14_ 40 breaths	72.8 (11.8)	75.5 (22.2)
IP_14_ 60 breaths	74.3 (12.8)	73.6 (19.7)
IP_14_ 80 breaths	74.4 (15.0)	73.9 (22.0)
IP_14_ loading	72.5 (10.8)^b^	74.4 (20.2)

*Note*: Data shown are Mean ± SD. All measures were analyzed using paired *t*‐tests.

Abbreviations: IP_14_, Inspiratory pressure at RPE14; IP_max_, maximal inspiratory pressure.

^a^ *p* < 0.01 significantly greater IP_14_ after 20 breaths than IP_14_ baseline;

^b^ *p* < 0.01 Significantly greater IP_14_ loading than IP_14_ baseline;

^c^ *p* < 0.01 Significantly greater IP_14_ baseline after CPAP.

To examine whether IP_14_ values are associated with OSA severity, the correlations between AHI and IP_14_ values at baseline and after loading were investigated before and after receiving CPAP treatment. Spearman's rho revealed a significant correlation between AHI and IP_14_ at baseline before patients received CPAP treatment (*r* = −0.567, *p* = 0.022). No correlations were observed between AHI and IP_14_ at baseline after CPAP treatment (*p* > 0.05). In addition, no significant correlations were detected with IP_14_ values after loading pre and post CPAP. Consequently, a lower inspiratory pressure generated by inspiratory muscles for an RPE14 at baseline was associated with higher AHI.

## 6 DISCUSSION

### 6.1 Main findings

In this study, we investigated the response of effort perception to fatiguing loading cycles of the inspiratory and leg muscle system in OSA patients and healthy subjects. The fatigue response was confirmed by increasing EMG amplitudes over the duration of a loading cycle and with increasing number of loading cycles in both muscular systems, that is, Intercostal muscles and quadriceps muscles. Fatigue responses were particularly apparent in OSA patients who revealed the largest increases in EMG amplitudes in both inspiratory and leg muscle systems during loading, corroborative of poor muscular endurance in both systems in OSA patients (Chien et al., 2010). Increased EMG amplitudes at the same force/pressure generation are shown to be a hallmark of peripheral muscular fatigue and further reflects the compensatory response by the neuromuscular system which attempts to maintain the work output (Stulen & De Luca, 1978). Our findings are consistent with previous studies reporting the concurrent loss of force generating capacity and progressive increase in quadriceps EMG amplitude following intermittent, submaximal loading protocols (Adam & De Luca, 2003; Bigland‐Ritchie et al., 1986; Carpentier et al., 2001).

Many factors have been proposed as potential cause of muscle activation changes from the central nervous system to the specific muscle groups. It has primarily been suggested that muscle activation changes are thought to arise as a consequence of increased recruitment of additional motor units with higher firing rates, alterations in the contractile properties of motor units, and potentially improved synchronization between discharge rates of motor units (Amann et al., 2010; Bigland‐Ritchie & Woods, 1984). However, the underlying muscle properties and neural responses are specific to the muscle group studied, loading stimulus and protocol adopted. Further research is required to focus upon the exact underlying mechanism. We additionally found the maximal leg muscle forces at baseline were much lower in OSA patients (on average 30%) compared with individuals with high levels of fitness confirming the declined muscular capacity/strength within this population.

For the inspiratory muscles, OSA patients used a higher proportion of accessory inspiratory muscle (trapezius) activation that is, % EMG amplitude, during the loading cycles compared with healthy individuals, suggesting that respiratory work is shared amongst several accessory muscles due to reduced primary inspiratory muscle capacity. Higher use of accessory respiratory muscles has been previously reported in obstructive conditions, OSA, and obesity (Cherniack & Guenter, 1961; O'Donnell et al., 2007; Sharp, 1976). In summary, our data showed that OSA patients in this study have a reduced muscular endurance and capacity in both, the respiratory and locomotor muscle systems, which agrees with earlier work. In addition, outcomes showed that the paradigm utilized in this study, successfully induced signs of peripheral fatigue in both muscular systems in groups with lower cardiovascular fitness and muscular strength.

Our main objective was to examine effort perception after repetitive fatiguing loading of the inspiratory and locomotor muscles in OSA patients and healthy individuals and its association with AHI in OSA patients. Our outcomes showed the load/pressure generated at a perceived effort following repeated bouts of loading was different for both inspiratory and leg muscles. In the healthy groups, loading of the quadriceps muscles did not alter the level of effort perception, demonstrated by stable isometric forces and EMG amplitudes generated at a perceived level of RPE14 (IF_14_). Interestingly, this was irrespective of the fatiguing loading cycles showing clear signs of increased peripheral fatigue (EMG amplitude increase during loading). This was also apparent in OSA patients who, however, did not compensate for the significant force generation losses induced by the fatigue protocol as demonstrated by lower isometric force values recorded at the effort level of 14 (IF_14_). However, the corresponding EMG amplitudes of the quadriceps revealed a slight elevation at IF_14_. The lower forces produced at IF_14_ in OSA patients can be interpreted as sustained peripheral fatigue which did not result in a correction of force during the task to generate forces matching the effort level of RPE14. This could be explained by a lack of integration of afferent feedback from force receptors in the generation of effort perception. As mentioned earlier, effort perception in the locomotor system is suggested to be independent of afferent feedback from force receptors and solely a result of corollary discharge of the motor command in the brain (Proske & Allen, 2019). However, other peripheral feedback systems such as chemoreceptor stimulation may also be implicated in limiting/interfering with the feedback signals of group III and IV afferents from the exercising limb muscles (Amann et al., 2009; Amann et al., 2010). Thus, the partial compensation of force production at a fixed effort perception may largely attributed to the muscle afferents in concert with other peripheral signals as a consequence of changing metabolic state of the limb muscle, resulting yet again in further feedforward /central motor command responses.

Furthermore, results also show that OSA patients have a reduced relative effort sensitivity for the leg muscles evidenced by using a significantly higher percentage of their muscular force capacity for the effort perception of somewhat hard/hard (RPE14). This suggests an adaptive response to the requirement of producing forces for locomotion during daily tasks, disregarding a high body weight in context with low muscular endurance and capacity. High effort sensitivity would impair task performance and would potentially lead to task avoidance, which has been shown in various populations with higher fatigability (Huebschmann et al., 2015; Williamson & Hoggart, 2005). In our study, OSA patients demonstrate outcomes in effort perception of the locomotor system expected for people with low muscular endurance and capacity, such as individuals with sedentary lifestyles, obesity, and/or clinical conditions, that is, chronic fatigue syndrome suggesting no specific alteration of effort perception for the locomotor muscles (Gandevia, 2001; Lloyd et al., 1991; Norregaard et al., 1995; Tomlinson et al., 2016; Zhu et al., 2007).

For investigating the effort perception of the inspiratory system in response to repetitive loading in OSA patients revealed rather different outcomes to the locomotor system. While the effort sensitivity seemed to be on similar levels in all groups at baseline, shown by no significant difference in the levels of IP_14_ values between groups, the changes in effort sensitivity in response to repetitive loading are profound. All groups reduced their effort sensitivity in response to repetitive loading, a significant increase in the inspiratory pressure generated at an effort level of somewhat hard/hard (IP_14_) was detected for all groups after loading. EMG analysis showed increasing EMG amplitudes during loading of Intercostal muscles, was particularly apparent in the OSA and low fitness groups. However, the increase in inspiratory pressure at RPE14 (IP_14_) from baseline to after loading was the highest in OSA patients revealing a strong desensitization of effort perception after loading (about 15% increase in IP_14_).

The principal finding in all groups showing an increase in inspiratory pressure generation for RPE14 in response to loading could be interpreted as an adaptive response to increased work for breathing (during loading cycles). Since effort perception is an integral part of dyspnoea sensation (Laviolette & Laveneziana, 2014), a reduction in sensitivity during repetitive loading would enable the avoidance of aversive feelings, that is, dyspnoea and possible concurrent task disengagement. Increased respiratory work and potential muscular fatigue are known from high ventilation rates during aerobic exercise (Dempsey et al., 2006); therefore, an adaptive alteration of effort sensitivity in the respiratory system would avoid early dyspnoea generation during exercise and tasks with higher ventilatory effort. The strongest reduction in effort sensitivity in response to repetitive loading in OSA patients, as seen in our study, supports this interpretation as an adaptive mechanism. Indeed, desensitization follows the order in the magnitude of fatigue during loading with larger increases in EMG amplitudes during loading sets in OSA over low fitness over high fitness, and fitness with high fitness group increasing the least followed by low fitness group and OSA patients in IP_14_ after loading. However, cardiovascular fitness could not be measured directly in OSA patients due to safety concerns.

With poor respiratory muscle endurance and capacity, effort‐related task disengagement could be avoided by reduction of respiratory effort sensitivity and by recruitment of accessory respiratory muscles (higher recruitment of Trapezius). Indeed, a lower sensitivity to respiratory loading was seen in OSA patients (Tun et al., 2000), as well as a lower detection threshold for restrictive flow in earlier work (McNicholas et al., 1984). However, the effort sensitivity IP_14_ at baseline was higher than expected for the low respiratory muscle endurance of inspiratory muscles; as mentioned before, OSA patients revealed a stronger EMG amplitude increase to repetitive loading than the healthy groups, suggesting peripheral fatigue. If the same rules would apply to the inspiratory muscle system as to the locomotor muscles, as seen in the limb muscle paradigm, it would be expected that OSA patients would use a higher percentage of their muscular (pressure) capacity for RPE14 at baseline; however, this was not detected. Moreover, the baseline IP_14_ values of OSA patients were significantly negatively correlated with AHI, revealing that OSA patients with higher inspiratory effort sensitivity at baseline were shown to have more severe obstructive sleep apnea with higher frequency of obstructive events and awakenings from sleep.

Furthermore, OSA patients revealed a pronounced change in their baseline IP_14_ values after 3 months of CPAP treatment shifting up to the level seen after repetitive loading and to a higher percentage of the maximal inspiratory pressure, with no association with AHI. This alteration resembles a move toward the expected values seen from the limb paradigm; poorer muscular endurance would change effort sensitivity towards lesser sensitivity using a higher percentage of their force/pressure capacity for RPE14. Consequently, although speculative, it is suggested that CPAP reversed the adaptive response which may contribute to higher frequency of obstructive events during sleep and could therefore be maladaptive and OSA‐specific.

On the contrary, the principal difference in the response of effort sensitivity to loading between the respiratory and limb muscle system, seen in the tested groups, reveals that effort perception is more flexible in the way it can integrate afferent feedback from receptors of various muscle systems. The limb muscle system seems robustly “neglecting” afferent receptor information about fatigue‐related force losses and seemingly only using corollary discharge for the effort perception, while the respiratory system appears to integrate afferent receptor feedback induced during the fatiguing loading cycles. An upregulation of motor command for a specific perception of effort within a system of various respiratory muscles (primary and accessory respiratory muscles) is only feasible if afferent feedback is used. (Figure 7a).

### 6.2 Methodological considerations

While this study provides further insight into the perceptual and muscular responses to loading in healthy male volunteers with different fitness levels, newly diagnosed OSA patients, and the effects of CPAP therapy, some limitations warrant discussion. First, we did not conduct sleep studies in our healthy group to exclude the possibility of OSA due to the limited resources available. Instead, we performed a series of sleep‐related questionnaires to highlight any signs/symptoms of OSA in our healthy groups. The Epworth Sleepiness Score (ESS) and Pittsburgh Sleep Quality Index (PSQI) were revealed to be significantly lower in both high and low fitness groups compared with OSA patients and classified as normal according to normative data (Buysse Charles et al., 1989; Johns, 1991). Therefore, we are confident the healthy participants recruited for the present study were not demonstrating signs/symptoms of OSA.

In spite of our best efforts to recruit healthy participants matched for age and weight we found the recruited OSA patients were considerably older and heavier than our high and low fitness participants. Future studies should attempt to recruit older and/or overweight participants without OSA in order to address whether age and weight differences exist. Furthermore, the current study did not assess female participants given the scarcity of newly diagnosed female patients with OSA within the sleep department at that present time. Future studies are required to determine whether sex differences within OSA exist in response to loading protocols. We also did not conduct an incremental exercise test to measure the maximal rate of oxygen uptake in OSA patients given the lack of medical supervision/assistance for testing patients with larger BMI and known cardiovascular risk factors. The CPAP treatment period of 3 months is considered short‐term when attempting to observe changes in the perceptual response to loading. Future studies should strive to examine the long‐term effect of CPAP therapy (>12 months) for further refinement of this study.

Finally, while surface EMG is an advantageous tool for measuring the amplitude of skeletal muscles during loading in a non‐invasive manner, the technique is limited by factors such as; noise, cross‐talk from nearby muscles, skin‐electrode contact (e.g., sweating), and thickness of subcutaneous fat (Reaz, Hussain, & Mohd‐Yasin, 2006). In order to reduce the influence of these factors we adopted a thorough cleaning procedure of the skin and followed standardized positions for electrode placement. Participants were further instructed not to excessively move the dominant leg during the loading protocol. We also followed standardized procedures by normalizing the EMG amplitudes according to the maximal RMS (% RMS) (Orozco‐Levi et al., 1995; Yokoba et al., 2003). Moreover, due to the interest for enabling a measure for load induced fatigue, we focused on the RMS of the EMG amplitude. However, an additional spectral analysis of the EMG signal could have given additional support in detecting signs of fatigue. A future spectral analysis could enable further insights in the process of fatigue in both muscular systems.

## 7 CONCLUSIONS

In summary, repetitive loading cycles revealed poor muscular endurance in both the limb and respiratory muscle system of OSA patients. Perception of effort showed different responses to inspiratory and limb loading protocols. In particular, the respiratory system of OSA patients was strongly downregulated with higher baseline sensitivity, which was correlated with AHI, and suggesting a possible maladaptive response of effort perception in OSA. Baseline respiratory effort sensitivity was reduced to post‐loading levels following 3‐months of CPAP treatment. Findings in the respiratory system of OSA and healthy populations suggest an active adaptive use of afferent feedback information for the adjustment of effort sensitivity. Future studies need to investigate the connection between neuronal systems relevant for effort perception and their influence on inspiratory force production during sleep in OSA.

## AUTHOR CONTRIBUTIONS

Claire Griffith‐Mcgeever: contributed to conceptualization and design of this study; collection, analysis, and interpretation of the data; and preparation of the manuscript. Hans‐Peter Kubis and Julian Owen: contributed to conceptualization and design of this study; interpretation of the data; and preparation of the manuscript. Christopher Earing and Damian McKeon: contributed to patient recruitment and acquisition of data. All authors read and approved the final manuscript.

## FUNDING INFORMATION

Claire Griffith‐Mcgeever received a Coleg Cymraeg Cenedlaethol PhD research scholarship (500497) in 2015.

## ETHICS STATEMENT

The study was approved by research ethics committees at School of Sport, Health and Exercise Sciences, Bangor University (P01‐16/17) and East Midlands‐Leicester South, Nottingham (17/EM/0162), conforming to the Declaration of Helsinki. Written informed consent was obtained from all participants before enrolling into the study.
